# Optimizing classroom environments for visually impaired school children a scoping review protocol

**DOI:** 10.1371/journal.pone.0308149

**Published:** 2024-10-17

**Authors:** Nur Aresya Ahmad Najmee, Zainora Mohammed, Mohd Harimi Abd Rahman, Norliza Mohamad Fadzil, Arimi Fitri Mat Ludin

**Affiliations:** 1 Optometry and Vision Sciences Program, Centre for Rehabilitation and Special Needs Study, Faculty of Health Sciences, Universiti Kebangsaan Malaysia Kuala Lumpur, Kuala Lumpur, Malaysia; 2 Biomedical Science Program and Centre for Healthy Aging & Wellness, Faculty of Health Sciences, Universiti Kebangsaan Malaysia, Kuala Lumpur, Malaysia; Xiamen University - Malaysia Campus: Xiamen University - Malaysia, MALAYSIA

## Abstract

Creating a universal and supportive learning environment is essential for the holistic education development of visually impaired school children. However, inadequate infrastructure, limited access to specialized tools, and a shortage of skilled teachers may contribute to lower self-confidence and academic performance. This underscores the necessity for specific guidelines and recommendations to address an optimal classroom setting that is tailored to their visual needs. In this paper, a scoping review protocol is introduced, utilizing the framework proposed by Arksey and O’Malley (2005), to address the following research questions: 1) Is there any established standard classroom setting for visually impaired school children? 2) What recommendations or specific guidelines exist regarding the physical measurement, layout, lighting, contrast, and appropriate position for visually impaired school children? The scoping review explored four electronic databases: PubMed, Scopus Emerald Insight, and Web of Sciences. The outcomes from the scoping review will offer conceptual elucidation, synthesize existing theoretical and empirical understandings, and propose the optimal classroom settings tailored to the visual needs of visually impaired schoolchildren. This paper introduces the a-priori study protocol, which outlines the planned methodology for conducting the scoping review in detail. This protocol has been officially registered with the Open Science Framework (OSF) at the following link: http://osf.io/z2sdt.

## Introduction

Childhood visual impairment is defined as irreversible loss of vision or a persistent visual impairment in children aged six to seventeen, that does not improve with refractive surgery or medical treatment [1]. In Malaysia, studies have revealed variability in the anatomical sites contributing to visual impairment, with retinal abnormalities accounting for 33% of cases [2], and the lens identified as the predominant site of visual loss in another study [3]. Many retinal disorders, whether idiopathic or genetic, frequently cause serious visual impairment or blindness. A study of children with genetic retinal diseases found that they had a lower quality of life than those with congenital cataracts, which may be corrected [4]. It highlights the importance of addressing individual needs based on specific anatomical factors affecting vision.

Childhood visual impairment can have long-term effects on education, mental health, social skills, and job opportunities [5]. Consequently, children with retinal problems often require special assistance in school, such as utilizing low vision aids and arranging modifications such as sitting in the front row, larger print materials, and better lighting. Given that many school administrators and management are willing to modify classroom settings for students with visual impairment, eye care specialists must make appropriate recommendations in order to create an environment that addresses the visual needs of students, supporting their academic success and improving their quality of life [6]. Adjustments and modifications aimed at enhancing the classroom environment can be implemented to accommodate students’ visual requirements. These may include modifications to lighting, such as adjustable or controllable lighting systems, as well as determining seating positions based on the severity of visual impairment to meet their specific needs effectively [7, 8].

Adjustable or controllable lighting systems offer flexibility in adjusting illumination levels to accommodate varying visual needs and preferences. For instance, low-vision students may benefit from brighter lighting to enhance contrast and visibility, while others may require softer lighting to minimize glare and visual discomfort. The minimum illumination required for visually impaired students in Malaysia is 400 lux to achieve optimal visual function. To enhance light levels, it is suggested to remove obstacles to natural light and add artificial lighting in special schools in Malaysia [9]

Moreover, determining seating positions based on the severity of visual impairment allows for enhanced clarity of the chalkboard and minimized visual strain to facilitate adaptation in mainstream educational settings. To ensure the teacher’s writing on the blackboard is easily readable from any point in the classroom, the board should be painted either black or green, with a primary reflectance factor of 15–30%. The glare index should be 16, and the illumination level should be set between 150–300 lux for both the blackboard and student desks inside the classroom [10]

Nevertheless, as of now, there are limited recommendations for architectural and visual aspects, encompassing classroom dimensions and capacity, layout, and contrast of materials that are specific to low-vision students to tailor their visual abilities. The allocation of space per student within educational buildings in Malaysia varies according to the type of school. In primary schools, the maximum norm of floor area per student ranges from 7.8 to 18.8 square meters, with preschools excluded from this range [11]. Meanwhile, secondary schools have a norm ranging from 10.5 to 21 square meters per student, yet, there is no specific capacity provision within standard classroom dimensions for visually impaired students. Furthermore, guidelines issued by the Department of Standards Malaysia and the Department of Public Works Malaysia aim to accommodate the general public and persons with disabilities, without specific considerations for visually impaired students [12, 13]. The development of guidelines and recommendations for classroom configurations addressing visual requirements for visually impaired students is crucial to ensure that the involvement and academic performance of these students are not hindered by suboptimal environments.

### Significance of the study

The review regarding classroom settings necessitates a comprehensive mapping and synthesizing of a wide range of literature, encompassing studies on teaching approaches, learning environments, educational technologies, and inclusive practices. This approach aids in identifying gaps and trends providing an overview that establishes the framework for future research projects and evidence-based improvements in educational settings. Research on inclusive education for visually impaired students is constrained [14]. Previous studies have examined the impact of indoor environments on their mobility [15] the creation of learning environments conducive to academic success [16, 17], as well as teaching strategies and approaches tailored to students with visual impairment [18]. However, to date there have been limited studies discussing the optimal classroom settings, considering furniture and seating arrangement, layout, lighting, color, and contrast, aimed at accommodating the unique visual needs and diverse spectrum of visual deficits of these students. Such considerations are crucial not only to help in mobility and navigation but also to enhance visual comfort.

The objective of the scoping review is to comprehensively identify and analyze pertinent peer-reviewed studies published in English, recognizing their established data analysis methodologies and academic credibility in addressing learning environments tailored to visually impaired schoolchildren. Additionally, the review will thoroughly examine relevant grey literature, including online dissertations, theses, and standard documents focused on classroom recommendations and modifications for visually impaired schoolchildren.

The synthesized findings from both peer-reviewed studies and grey literature will serve as invaluable resources in a structured manner for policymakers and researchers striving to design inclusive classroom settings tailored to the unique needs of visually impaired schoolchildren.

This paper serves as an a-priori-developed study protocol, providing a thorough description of the intended technique for carrying out the scoping review. Before conducting the review, a study protocol must be written; however, for scoping reviews, publishing is optional. The publication aims to reduce reporting bias and promote transparency [19].

## Methods

Scoping reviews offer particular advantages in investigating new research areas and gaps, as well as elucidating fundamental principles. Considering the objective to comprehensively examine the empirical studies focusing on classroom recommendations and modifications, designed to create an optimal learning environment for visually impaired schoolchildren, addressing their unique visual needs, a decision has been made to conduct a scoping review. In order to ensure transparency and clarity throughout the process, meticulous documentation of the research objectives and methods will adhere to the protocol delineated in this paper. To enhance the systematic process, the study has adopted the PRISMA-P (Preferred Reporting Items for Systematic Review and Meta-Analysis Protocols) 2015 checklist, refer to Supporting Information (S1 File).

The scoping review will be conducted following the framework presented by Arksey and O’Malley [20] and the methodology provided by Joanna Briggs Institute JBI aimed at evaluating the justification for undertaking a comprehensive review [21, 22].

This recommendation is particularly pertinent as scoping analyses play a crucial role in addressing broader questions, encompassing both quantitative and qualitative literature with minimal restrictions, thereby allowing for the inclusion of a diverse range of evidence sources [23].

A detailed description of ethical permission and disclosure of any conflict of interest should be provided. This study protocol is part of a scoping review for a broader study focusing on visual profiling and classroom settings to meet the visual and academic needs of visually impaired students. The overarching study has been approved by Universiti Kebangsaan Malaysia, with ethics permit JEPUKM_JEP-2023-470.

### Research questions

1. What specific classroom settings are tailored to accommodate the individual visual needs of visually impaired school children?
2. What are the recommendations or guidelines for physical measurement, layout, lighting, contrast, and appropriate position for visually impaired school children?

### Research sub questions

1. How can the configuration of the classroom environment be optimized to improve mobility and navigation for visually impaired students, considering the diverse spectrum of visual deficits?
2. How do lighting, color, and contrast influence the perceptual clarity and comprehension of material to meet the individual visual needs of visually impaired students?

### Inclusion and exclusion criteria

The visually impaired school children and their classroom setting are the main focus of this study.

#### Participants

The study population comprises visually impaired or low-vision schoolchildren aged 7 to 18 years old. Participants with other sensory impairments, multiple morbidities, or who fall outside the age range will be excluded from consideration.

#### Concept

This scoping review will include qualitative, quantitative, and mixed-method peer-reviewed journals, theses, dissertations, conference proceedings, acts, and standard documents published between 2000–2024 in English and Bahasa Melayu. Including acts and standard documents as grey literature will facilitate a holistic understanding of classroom recommendations and modifications for visually impaired schoolchildren, capturing innovative approaches and real-world practices that may not yet be reflected in peer-reviewed literature. A rigorous critical appraisal process will accompany it to ensure the quality and relevance of the gathered information. These publications will involve mainstream and specialized educational settings across various geographical locations and educational systems.

#### Context

Eligible studies in this review are studies that investigate or discuss interventions related to lighting, contrast, physical measurement, layout, and seating position in classrooms for visually impaired students. Additionally, studies assess the impact of classroom settings on the educational experience, participation, and academic outcomes of visually impaired students, and studies report on the preferences and feedback of visually impaired students regarding classroom design. However, studies focusing on low-vision aids and assistive technologies designed to optimize the classroom environment for visually impaired students will be excluded. This decision aims to maintain a focused examination of the organizational aspects and visual comfort within the physical classroom environment, ensuring a direct exploration of their impact on these students.

### Determination of search strategy

In determining the search strategy, two independent reviewers will conduct a comprehensive survey of literature related to classroom settings for visually impaired children, matching specified terms.

To initiate the search, a set of terms was employed, including ’school child*,’ ’student*,’ ’learner,’ ’visually impaired,’ ’low vision,’ ’classroom,’ ’size,’ ’dimension,’ ’lighting,’ ’illumination,’ ’seating,’ and ’contrast.’ These terms were grouped to form a general query using Boolean operators in Alma’s advanced search feature, specifically utilizing ’OR’ and ’AND.’ An exemplar of the general query is as follows: (’school child*’ OR ’student*’ OR ’learner,’) AND (’visually impaired’ OR ’low vision’) AND ’classroom’ AND (’size’ OR ’dimension’ OR ’lighting’ OR ’illumination’ OR ’seating’ OR ’contrast’). The general query was adapted to adhere to the search guidelines of each electronic database, which included applying filters such as language, search dates, and document types (e.g., journal articles, and conference proceedings). Ultimately, four search queries were generated. The outcome of each search’s result will be recorded in the Endnote Web library database. The example of a full literature search encompasses manual searching and screening of reference lists, which can be seen in Supporting Information (S2 File).

The literature searches were performed across four databases: PubMed, Scopus, Emerald Insight, and Web of Sciences. These databases were selected because they include multidisciplinary fields such as health sciences, education, and architectural journals (eg., Journal of Visual Impairment & Blindness, Indian Ophthalmology, PLoS One, and Frontiers of Architectural Research).

### Study selection

After the initial search, two authors from the research team will autonomously select studies based on the title and abstract adhering to the predefined inclusion and exclusion criteria. The resulting studies need to be categorized into five categories; qualitative research, randomized controlled trials, non-randomized studies, quantitative descriptive studies, and mixed [24]. Articles meeting the inclusion criteria will be retrieved in full, and their details will be incorporated using the systematic review tool Rayyan.ai. The same tool will be independently used by two authors, specifically to assess the eligibility of the studies and ensure alignment with the research questions and objectives. Any disagreements among the authors will be resolved through consensus, and if necessary, a third researcher will mediate any remaining discrepancies or decisions. Excluded studies will be categorized based on criteria such as incorrect setting, population, intervention, and outcome.

### Charting the data

The charting form to be used in the scoping review will be adapted based on the templates proposed by Arksey and O’Malley [20], supplemented by the guidelines from Levac, Colquhoun, and O’Brien [25]. In scoping reviews, charting serves to establish consensus on the essential information to be extracted from each study. Consequently, studies will be deconstructed into data within an extraction form, capturing the following key components:

*Article title; Authors; Publication year; Country; Population; Settings (E*.*g*.: *school*, *hospital*, *university*); *The aim of the study; Analysis type; Study design; Research instruments/variables; Intervention; Recommendation and Modification of classroom setting; Context of research (local*, *regional*, *national*, *transnational); Results/findings* (*Outcome*).

The planned schedule and researcher participation in the scoping study are outlined in the Supporting Information (S3 File), with the study targeted for completion within 8 months.

### Presentation of results

The outcome will be presented in tabular format, accompanied by a narrative summary aligned with the objectives of the current scoping review. A data presentation table will be generated based on the extracted data, organized by study type. The decision-making process for the review will be illustrated through a flow chart, employing the A PRISMA-ScR flow chart methodology [23], encompassing search results from various databases and sources, the elimination of duplicate citations, stages of study selection (title/abstract and full text), reasons for excluding papers post-full-text reading, and the final count of studies included researchers. Refer to S4 File for the search flowchart following PRISMA guidelines.

## Limitations

The purposeful investigation is constrained by limitations. Due to human and time resources, the search of databases has been restricted to a specific number. Consequently, any data that is not indexed in these databases will not undergo processing. The search articles and documents are also limited to full text in either English or Bahasa Melayu language. Hence, publications in other languages will therefore not be considered. Moreover, the evaluation of the record’s quality will not be conducted, potentially resulting in the inclusion of studies and publications with deficiencies in their design.

## Discussion

Uncorrected refractive errors, classroom overcapacity relative to size, inadequate illumination, and the size of learning materials can all affect students’ perception of information and contribute to visual impairment [26]. The inclusion of visually impaired schoolchildren in education is primarily influenced by the classroom environment which can impact their visual integration and comfort levels [27, 28]. Establishing an inclusive learning environment is crucial, especially during the formative school years, as schools play a vital role in preparing visually impaired students to navigate challenges they may encounter in the broader world [29]. Moreover, studies have suggested that proper modifications can enhance both academic and social experiences for visually impaired students and considering their visual abilities and needs can prepare them for mainstream settings [6, 27, 28, 30]

Simple modifications, such as adjusting the teacher’s handwriting size and seating arrangement, can assist visually impaired students with their visual tasks in the classroom, thereby reducing visual stress. Minor changes in seating arrangements and classroom layout can promote better engagement among special students while fostering independence [31]. Overall, creating an optimal classroom not only facilitates the movement of these students within the built environment but also promotes a friendly design that enhances their use and enjoyment of the space.

Nonetheless, despite the existence of classroom modification and adjustment for visually impaired students in school practices, to date, there appears to be a lack of comprehensive effort to systematically synthesize these resources. This gap underscores the need for a structured approach to gather, analyze, and disseminate relevant information. Therefore, the planned scoping review aims to synthesize existing classroom setting modifications and recommendations, serving as invaluable references for policymakers and researchers striving to design inclusive classroom settings tailored to the unique needs of visually impaired students.

By employing a scoping review protocol, this effort ensures a structured and transparent method for gathering, analyzing, and disseminating relevant information. The systematic synthesis of existing resources not only enhances the reliability and applicability of the findings but also provides practical guidance for creating inclusive learning environments for visually impaired students, thus bridging the gap between research and practice in this critical area.

Furthermore, the sustainability impact of the study extends globally by promoting awareness and advocacy for inclusive education by disseminating the outcome through academic publications, conferences, and professional networks. This can inspire further research and action in the field of inclusive education on a global scale. This ripple effect can lead to sustained efforts to improve educational opportunities and outcomes for visually impaired students worldwide, contributing to a more sustainable and equitable future for all.

## Supporting information

S1 File PRISMA-P (Preferred Reporting Items for Systematic Review and Meta-Analysis Protocols), 2015 checklist. (DOC)

S2 File Example of database search. (DOCX)

S3 File Study schedule. (DOCX)

S4 File A PRISMA-ScR flow chart. (TIF)
