# Supplementary material for: Optimizing classroom environments for visually impaired school children a scoping review protocol
Source: PLoS One. 2024 Oct 17;19(10):e0308149. doi: 10.1371/journal.pone.0308149 (PMC11486386; doi:10.1371/journal.pone.0308149)
Supplement: S2 File — (DOCX) [file pone.0308149.s002.docx]

**Appendix S2: Example of Database Search**

| Database | No of articles | Search String |
| --- | --- | --- |
| Pubmed | 28451 | school child* or pupil* or student* or learner* AND visually impaired* or visual dysfunction* or blind* or visual disable* or impaired vision or vision disorder* or vision disability* or vision deterioration* or vision loss or visual defect* or abnormal vision or low vision or reduced vision or partial sight* or near blind or subnormal vision or diminished vision or ocular dysfunction* or ocular disorder* AND "classroom size*" or "classroom dimension*" or "classroom measure*" OR "classroom layout*" or "classroom design" or "classroom organization*" or "classroom setting" OR "classroom light*" or "classroom illumination" OR classroom contrast" OR seating |
| Web of Science | 381 | (((((((((((((((((((((((((((((((((ALL=(student)) OR ALL=(school child*)) OR ALL=(pupil)) OR ALL=(learner)) AND ALL=(visual impaired )) OR ALL=(visual dysfunction)) OR ALL=(low vision)) OR ALL=(blind)) OR ALL=(visual disable)) OR ALL=(vision loss)) OR ALL=(impaired vision)) OR ALL=(vision disorder)) OR ALL=(vision deterioration)) OR ALL=(visual disability)) OR ALL=(vision loss)) OR ALL=(reduced vision)) OR ALL=(partial sight)) OR ALL=(subnormal vision)) OR ALL=(diminished vision)) OR ALL=(ocular dysfunction)) OR ALL=(ocular disorder)) AND ALL=(classroom size)) OR ALL=(classroom dimension)) OR ALL=(classroom layout)) OR ALL=(classroom measure)) OR ALL=(classroom layout)) OR ALL=(classroom design)) OR ALL=(classroom organization)) OR ALL=(classroom settings)) OR ALL=(classroom lighting)) OR ALL=(classroom illumination)) OR ALL=(classroom contrast)) OR ALL=(contrast materials)) OR ALL=(classroom seating) |
| SCOPUS | 259 | student OR school child* OR pupil OR learner AND visual impaired OR visual dysfunction OR low vision OR blind OR visual disable OR vision loss OR impaired vision OR vision disorder OR vision deterioration OR visual disability OR vision loss OR reduced vision OR partial sight OR subnormal vision OR diminished vision OR ocular dysfunction OR ocular disorder AND classroom OR size OR dimension OR layout OR design OR organization OR lighting OR illumination OR contrast OR seating |
