# Supplementary material for: Optimizing classroom environments for visually impaired school children a scoping review protocol
Source: PLoS One. 2024 Oct 17;19(10):e0308149. doi: 10.1371/journal.pone.0308149 (PMC11486386; doi:10.1371/journal.pone.0308149)
Supplement: S3 File — (DOCX) [file pone.0308149.s003.docx]

**Appendix S3: Study Schedule**

| Stage | Task | Month | Status | Accountable Person |
| --- | --- | --- | --- | --- |
| 1 | Identifying the research question | 09/2023 | Done | AN, AF, ZM |
| 2 | Protocol and registration | 10-11/2023 | Done | AN, AF, ZM, NF, HR |
| 3 | Identifying relevant studies | 11-12/2023 | Done | AN, ZM, NF |
| 4 | Studies selection | 12/2023 | Done | AN, ZM, NF |
| 5 | Charting the data | 12-01/2024 | In process | AN, ZM, AF |
| 6 | Collecting, summarizing, and reporting the results | 02-04/2024 | Open | AN, AF, ZM, NF, HR |
