# Supplementary figures and images for: Optimizing classroom environments for visually impaired school children a scoping review protocol
Source: PLoS One. 2024 Oct 17;19(10):e0308149. doi: 10.1371/journal.pone.0308149 (PMC11486386; doi:10.1371/journal.pone.0308149)

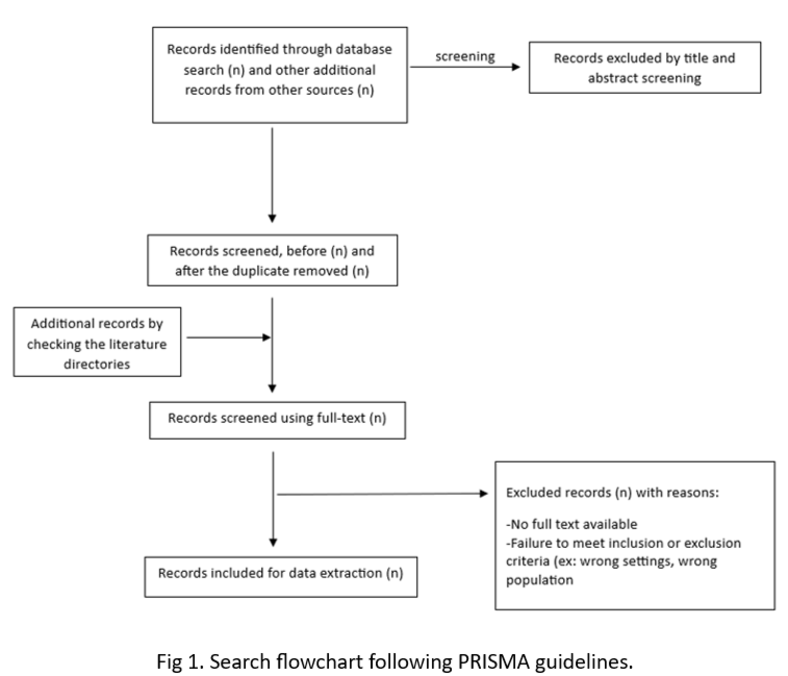

Supplement: S4 File — (TIF) [file pone.0308149.s004.tif]
